# From Good Intentions to Proven Interventions: Effectiveness of Actions to Reduce the Health Impacts of Air Pollution

**DOI:** 10.1289/ehp.1002246

**Published:** 2010-08-20

**Authors:** Luisa V. Giles, Prabjit Barn, Nino Künzli, Isabelle Romieu, Murray A. Mittleman, Stephan van Eeden, Ryan Allen, Chris Carlsten, Dave Stieb, Curtis Noonan, Audrey Smargiassi, Joel D. Kaufman, Shakoor Hajat, Tom Kosatsky, Michael Brauer

**Affiliations:** 1 School of Human Kinetics, University of British Columbia, Vancouver, British Columbia, Canada; 2 British Columbia Centre for Disease Control, Vancouver, British Columbia, Canada; 3 Swiss Tropical and Public Health Institute, Basel, Switzerland; 4 University of Basel, Basel, Switzerland; 5 International Agency for Research on Cancer, Lyon, France; 6 Cardiovascular Epidemiology Research Unit, Beth Israel Deaconess Medical Center, Harvard Medical School, Boston, Massachusetts, USA; 7 Division of Respiratory Medicine, University of British Columbia, Vancouver, British Columbia, Canada; 8 Providence Heart and Lung Institute, St. Paul’s Hospital, Vancouver, British Columbia, Canada; 9 Faculty of Health Sciences, Simon Fraser University, Burnaby, British Columbia, Canada; 10 School of Environmental Health and; 11 Department of Medicine, University of British Columbia, Vancouver, British Columbia, Canada; 12 Population Studies Division, Healthy Environments and Consumer Safety Branch, Health Canada, Ottawa, Ontario, Canada; 13 Department of Epidemiology and Community Medicine, University of Ottawa, Ottawa, Ontario, Canada; 14 Center for Environmental Health Sciences, University of Montana, Missoula, Montana, USA; 15 Département de Santé Environnementale et Santé au Travail, Université de Montréal, Montréal, Quebec, Canada; 16 Institut National de Santé Publique du Québec, Montréal, Quebec, Canada; 17 Department of Environmental and Occupational Health Sciences, University of Washington, Seattle, Washington, USA; 18 London School of Hygiene and Tropical Medicine, University of London, London, United Kingdom

**Keywords:** air pollution, antioxidant, cardiovascular, exposure, intervention, public health, respiratory, urban planning

## Abstract

**Background:**

Associations between air pollution and a multitude of health effects are now well established. Given ubiquitous exposure to some level of air pollution, the attributable health burden can be high, particularly for susceptible populations.

**Objectives:**

An international multidisciplinary workshop was convened to discuss evidence of the effectiveness of actions to reduce health impacts of air pollution at both the community and individual level. The overall aim was to summarize current knowledge regarding air pollution exposure and health impacts leading to public health recommendations.

**Discussion:**

During the workshop, experts reviewed the biological mechanisms of action of air pollution in the initiation and progression of disease, as well as the state of the science regarding community and individual-level interventions. The workshop highlighted strategies to reduce individual baseline risk of conditions associated with increased susceptibility to the effects of air pollution and the need to better understand the role of exposure duration in disease progression, reversal, and adaptation.

**Conclusion:**

We have identified two promising and largely unexplored strategies to address and mitigate air pollution–related health impacts: reducing individual baseline risk of cardiovascular disease and incorporating air pollution–related health impacts into land-use decisions.

Associations between air pollution and multiple health effects are now well established (Pope 2007; Pope and Dockery 2006; Pope et al. 2002). For key pollutants such as particulate matter (PM) and ozone (Green et al. 1999), there are no established thresholds of exposure below which population health impacts are absent. Given that everyone is exposed to some level of air pollution, the attributable health burden can be high, particularly for vulnerable subpopulations. Recent evidence that air pollution leads to inflammatory processes that mediate a variety of diseases suggests an expanding range of health impacts related to air pollution exposure. Consequently, the population health burden may be greater than previously believed.

A discussion of the biological mechanisms by which air pollution leads to cardiovascular and respiratory disease has been covered in detail elsewhere (Brook et al. 2010; Ko and Hui 2009; Mittleman 2007; Nogueira 2009; Patel and Miller 2009) and is beyond the scope of this review. However, a mechanistic understanding provides information on the effects of timing and exposure duration on disease development and progression, how pollutants interact with other stressors, and potential mitigating factors such as nutritional supplementation or medications. Ambient PM affects respiratory and cardiovascular disease development and exacerbation via pulmonary (neurologic) reflexes and pulmonary inflammation. Under some circumstances, these responses result in systemic inflammation, oxidative stress, and altered vascular function. Collectively, these processes can contribute to cardiovascular and pulmonary diseases, including atherosclerosis.

Evidence from natural experiments (Clancy et al. 2002; Parker et al. 2008; Pope 2007) and from analyses of long-term trends (Laden et al. 2006; Pope et al. 2009) indicates that reducing air pollution has clear health benefits. Traditionally, air quality management has focused on emissions-based pollution control. Although regulations promoting cleaner vehicle engine technology, power production, and industrial combustion processes have clearly led to decreased emissions, increases in vehicle-kilometers traveled and overall power generation and industrial activity may offset their effectiveness. Interventions separating people from pollution, which reduce exposure independent of emissions controls and mitigate health impacts, have largely been overlooked as components of formal strategies. For example, land-use decisions typically do not consider air pollution–related health impacts and do not require minimum distances between sources and individuals. A consequence of this has been the siting of residences, schools, and hospitals near major traffic arteries. Modification of the infiltration of outdoor pollutants into indoor environments, which is largely a function of air exchange and building design, offers further opportunities for exposure reduction. Activity modification at an individual level such as altering the duration, intensity, and location where individuals are physically active can also help reduce air pollution exposure and dose. Because the benefits of exercise on health are well established, whereas the net consequences of physical activity in polluted environments remain unclear, recommendations on this topic must carefully weigh the benefits and risks of outdoor physical activity. To make further progress in reducing air pollution–related health impacts, a new framework is needed that incorporates strategies at regulatory, community, and individual levels, to reduce both emissions and exposures.

## Objectives

An international workshop with participants from multiple disciplines, titled “From Good Intentions to Proven Interventions: Effectiveness of Actions to Reduce the Health Impacts of Air Pollution,” was convened 26–27 March 2009 in Vancouver, British Columbia, Canada. Participants included health and environmental researchers, public policy makers, air quality managers, health care providers, and nongovernmental organization representatives. The primary focus of the workshop was to discuss evidence of the effectiveness of actions, at the community and individual level, to reduce health impacts of air pollution. Although national-level policies and management programs as well as specific emissions reductions programs have received considerable attention, this workshop focused on the state of the science around specific community- and individual-level interventions. Some examples are behavior and its impact on exposure, medications and their influence on health outcomes given air pollution exposure, and strategies to reduce individual baseline risk of conditions associated with increased susceptibility to the effects of air pollution. The current understanding of biological mechanisms such as systemic and local inflammatory impacts and the role of air pollution relative to these disease processes, were discussed, as was the role of exposure duration in disease initiation progression and reversal.

## Community-Level Interventions to Reduce Exposure

Past and current outdoor air quality management activities have largely focused on emissions reductions. From a public health perspective, activities such as technological improvements in combustion technology or fuel standards have the advantage of avoiding the well-documented challenges of persuading individuals to voluntarily change their behavior in order to protect themselves. Air pollution controls have resulted in substantial decreases in levels of air pollution, leading to measurable health benefits (Clancy et al. 2002; Downs et al. 2007; Medley et al. 2002; Pope et al. 2009; Schindler et al. 2009). Analysis of the effectiveness of regulations and air quality management actions to reduce air pollution and its associated health impacts is an active area of research that is discussed in more detail elsewhere (van Erp et al. 2008). Targeting specific sources that contribute significantly to air pollution is an important strategy in improving air quality. During the workshop, several case studies of community-level interventions were presented including Dublin’s (Ireland) ban on sales of coal, Libby’s (Montana) woodstove exchange program, and London’s (United Kingdom) congestion charge zone (CCZ) and low-emission zone (LEZ) programs. These were examples of how source substitution, technology upgrades, and urban/transportation planning can lead to decreases in exposure and/or health impacts. Although we briefly review these examples below, it is important to consider that community-level efforts will largely be location specific because they focus on major sources of local pollution.

Before the 1990 ban on the sale of coal, coal-related combustion was a major source of ambient air pollution in Dublin (Clancy et al. 2002). Within 6 years of the ban, ambient levels of black smoke and sulfur dioxide decreased 70% and 35%, respectively. During the initial 72-month period after the ban, total mortality decreased by 6% [95% confidence interval (CI), 4–7%] (Clancy et al. 2002). As expected, the largest effects were seen for respiratory and cardiovascular deaths, with decreases of 16% (95% CI, 12–19%) and 10% (95% CI, 8–13%), respectively.

In many communities, residential wood heating is an important contributor to wintertime pollution (Naeher et al. 2007). Because wood burning typically occurs in residential areas, the intake fraction (the ratio of the total mass of a pollutant inhaled to the mass of the pollutant emitted) of woodsmoke PM with an aerodynamic diameter of ≤ 2.5 μm (PM_2.5_) is higher compared with PM_2.5_ from other sources, such as traffic (Ries et al. 2009); this suggests that reducing woodsmoke emissions can effectively reduce PM_2.5_ exposures. To address this, woodstove exchange programs that encourage residents to replace older stoves with newer, cleaner burning models have been broadly implemented. Follow-up of a large woodstove exchange campaign in Libby, where nearly 90% of woodstoves were replaced or removed, showed reductions of 20%, 64%, and 50% in average wintertime concentrations of (November–February) PM_2.5_, polycyclic aromatic hydrocarbons, and levoglucosan (a woodsmoke tracer), respectively, compared with precampaign levels (Bergauff et al. 2008; Ward et al. 2009). Preliminary analysis across the four winter seasons spanning the stove exchange found decreased reporting of childhood wheeze [odds ratio (OR) = 0.75 per 5-μg/m^3^ reduction in ambient PM_2.5_; 95% CI, 0.56–1.00], upper respiratory infections, and bronchitis.

Traffic-related air pollution is an important source of primary and secondary pollutants and a major contributor to intraurban variability in pollutant concentrations. Accordingly, urban and transportation planning approaches may be effective in reducing this variability, as well as the overall impact of this pollution source. To address traffic congestion in central London, a congestion charge scheme (CCS) was implemented in 2003. Although the objective of the CCS was to reduce the traffic congestion in the central city zone, air pollution reduction was a potential co-benefit (Tonne et al. 2008). The implementation of the CCS was estimated to lead to a modest decrease of 0.73 μg/m^3^ in the annual average nitrogen dioxide concentration in the CCZ; however, this decrease was associated with an increase of 183 years of life gained per 100,000 population in the CCZ (Tonne et al. 2008). Although traffic congestion decreased initially, levels did reach pre-CCS conditions after 4 years, which may account for the limited improvement in air quality. Other positive health impacts that may have resulted from the implementation of the CCS, but were not formally investigated, included an increase in active transport (cycling and walking, albeit mitigated by exposure in a high-traffic zone), a decrease in noise pollution and related stress, and a decrease in vehicle traffic accidents. More recently, London has also implemented an LEZ specifically to target air pollution. The most polluting vehicles (as defined by the program), including heavy-duty diesel vehicles, larger vans, and buses, must pay a daily charge to travel through the LEZ (Kelly and Kelly 2009). By 2012, it is estimated that this program will lead to a 6.6% reduction in PM_10_ (PM with aerodynamic diameter ≤ 10 μm) levels (Kelly and Kelly 2009). LEZ programs have also been implemented elsewhere, including Tokyo, Japan, and Sweden, to reduce air and noise pollution.

The examples above illustrate how targeted interventions can affect sources such as woodsmoke and vehicle traffic that operate in close proximity to populations. Vehicle traffic has become a major source of air pollution in many communities, and the benefits of strategies that address only emissions reductions through improved vehicle technology and fuel quality may be at least partially offset by increases in vehicle-kilometers traveled. Although the future introduction of zero-emission vehicles such as electric cars may eliminate the need for alternative approaches to reduce the impact of traffic-related air pollution, the broad proliferation of vehicles that do not emit pollutants is likely to be at least 20–30 years away (America’s Energy Future Panel on Energy Efficiency Technologies 2009). Until zero-emission vehicles are universal, land-use planning that favors sprawl and increased commuting distances, coupled with poor public transit options, places greater dependence on vehicle travel. This is likely to result in increased emissions of ozone precursors as well as increased pollutant exposure for those residing in areas close to major traffic arteries, sitting in vehicles during heavy traffic, and attempting to walk, run, or bike along roads. Even in a scenario of decreasing per-vehicle emissions, a global economy that relies heavily on the transport of goods over large distances (Perez et al. 2009) and increasing industrial and transportation activity close to communities (Hricko 2008) may also result in elevated exposures. A paradigm shift in how cities are designed and organized, with separation of densely populated areas from major traffic arteries coupled with continued emissions reduction (e.g., via low-emission public transit) could significantly reduce individual exposure to traffic-related air pollutants. Such planning approaches must also consider other relationships between built environment and health, such as the relationship between increased sprawl and ozone concentrations (Stone 2008) and between increased neighborhood walkability (via higher density, street connectivity, and mixed-use design) and elevated concentrations of traffic-related pollutants (Marshall et al. 2009).

Although individuals are not typically exposed to single pollutants, experimental and epidemiologic study designs are limited in their ability to investigate the health effects of concurrent exposure to multiple pollutants. This is also a limitation of current air quality management. From a regulatory standpoint, guidelines, standards, and emissions limits are developed for single pollutants, under the notion that a given pollutant, although present in mixtures, acts individually to affect health and the environment (Nadadur et al. 2007) and serves as a proxy for a more complex exposure. Although it is challenging to implement regulatory approaches for pollutant mixes, a deviation from the current single-pollutant approach (Dominici et al. 2010) needs to be considered. Synergy involving ozone and other pollutants such as black carbon, and nitrogen dioxide has been demonstrated in animal and human populations (Mauderly and Samet 2009). In combination, these pollutants may cause a greater additive effect on lung function, cytokine production, and cardiac output and stroke volume compared with the individual pollutants themselves (Mauderly and Samet 2009). Assessing the effects of individual pollutants likely presents only a partial description of the overall health impact of air pollution exposure.

Given the potential for additive or synergistic effects, approaches that reduce exposure to multiple pollutants may be more effective than efforts to reduce emissions of single compounds. For example, with accumulating evidence of cardiopulmonary morbidity and mortality associated with traffic-related air pollution exposure (Health Effects Institute 2010), increasing the distance between populations and major roads may result in substantial health benefits. Attenuated lung function, increased markers of oxidative stress, and airway inflammation have been shown in asthmatics and nonasthmatics exposed to road-traffic pollution (Barraza-Villarreal et al. 2008; McCreanor et al. 2007; Romieu et al. 2008a). Additionally, children exposed to higher levels of traffic-related pollution (> 0.41 μg/m^3^ elemental carbon attributable to traffic) before 12 months of age have an increased risk of persistent wheeze (OR = 1.75; 95% CI, 1.07–2.87) (Ryan et al. 2009). Individuals residing within 50 m of a major road have a 63% excess risk of developing high coronary artery calcification compared with those living > 200 m away from a major road (Hoffmann et al. 2007), suggesting that the separation of residential areas from major roads may be an important public health intervention. Although separating individuals from pollution sources is likely to have health benefits, not all pollutants (i.e., ozone) decrease with increasing distance from roadways (Beckerman et al. 2008).

More specific evidence of the benefits related to interventions can be found in recent research indicating that adults who moved away from residences in close proximity to traffic (< 150 m from a highway or < 50 m from a major road) had a lower risk of coronary heart disease (CHD) mortality than did those remaining in locations close to traffic; in both cases, the risk of CHD mortality was greater than in those who never lived in close proximity to traffic (Gan et al. 2010). Additionally, children who moved away from residences with high background PM_10_ experienced an increased rate of lung function growth compared with children who moved to areas with high PM_10_ (Avol et al. 2001). Finally, introducing an electronic toll collection along the New Jersey Turnpike to reduce congestion was associated temporally and spatially with reductions in incidence of prematurity and low birth weight among mothers living near toll plazas (Currie 2009). These findings suggest that community design that aims to separate such facilities as schools, child care centers, and hospitals from major traffic arteries or approaches to mitigate traffic congestion can reduce exposure and impacts among vulnerable members of the population (California Air Resources Board 2005; Ministry of Environment 2006a, 2006b). In addition to simple separation of populations from traffic arteries, the establishment of “mixed-use” (i.e., residential, commercial, recreational) and high-density areas would allow for more “walkable” cities, potentially leading to reduced emissions through decreased automobile use. “Walkability,” a measure of how conducive an environment is to walking, can help to predict levels of physical activity and active transportation within the community (Frank and Engelke 2005). Community design facilitating improved fitness may reduce the health impacts of air pollution by decreasing the proportion of the population with underlying cardiopulmonary disease risk factors, and therefore reduce their susceptibility to air pollution. Therefore, the development of more walkable communities has the potential to provide population-level health benefits through both lower emissions and a decrease in underlying cardiovascular risk.

## Interventions Directed to Individuals

In addition to the air quality management strategies focused on emissions reductions and local initiatives to control sources and separate them from residential locations, schools, and health care facilities, the workshop highlighted the value of lowering baseline health risks to reduce pollution-related health impacts. Specifically, the implementation of established primary, secondary, and tertiary interventions (e.g., controlling hypertension, lowering lipids, reducing obesity, promoting physical activity and smoking cessation) for diseases affected by air pollution exposure will serve to reduce the overall burden of disease associated with air pollution exposure. For example, sedentary individuals and those with a diet deficient in antioxidants or with a high salt diet may have an increased risk of developing cardiovascular disease (Marchioli 2003; Qin et al. 2009; Warburton et al. 2006) and may therefore also be more susceptible to the effects of air pollution. Through diet modification, exercise, and possibly via antioxidant supplementation, individuals can potentially reduce their personal susceptibility. Several of these approaches are discussed in more detail below.

### Exercise

In most developed countries, both air pollution and physical inactivity pose significant health risks to urban populations. The benefits of regular exercise on cardiovascular disease incidence and progression are unequivocal (Warburton et al. 2006). Active transportation (e.g., walking or cycling to work) can increase physical activity and reduce the burden of cardiopulmonary disease; however, the health benefits of active transport may be partially compromised if location and potential air pollution exposure are not also considered. Exercise leads to increased PM inhaled dose (Panis et al. 2010) and, under some circumstances, to increased PM deposition (Daigle et al. 2003). Further, outdoor exercise among children who live in areas with high levels of ozone has been associated with an increased risk of asthma development (McConnell et al. 2002). Initial analyses suggest that although walkable areas may have lower levels of ozone and therefore provide multiple health benefits, they may also promote higher exposures to primary traffic-related pollutants (Marshall et al. 2009). Furthermore, the design of many communities represents a challenge to promoting physical activity because of proximity to pollutant sources near residential and recreational areas, lack of sidewalks, and long commuting distances. Given this apparent paradox, it is imperative to better understand the interaction of exercise and poor air quality on cardiorespiratory health and function.

Much attention has been paid to “smart growth” that reduces community dependence on the automobile and, in so doing, promotes physical activity and reduces pollutant emissions. The important public health impacts related to air pollution exposure suggest a need for even smarter growth that focuses on health promotion while also considering air pollution exposure. Examples include active transportation “green” corridors that are separated from major traffic arteries, design of neighborhoods that are both walkable and high density, serving communities with mass transit, and incentives to reduce emissions in urban centers. Further, the development of urban design and transportation planning tools that incorporate health-promoting attributes and that reduce individual-level exposure during travel are needed (e.g., Su et al. 2010; University of British Columbia 2010).

### Nutrition

In addition to increasing physical activity, dietary changes can reduce the risk of disease development and therefore reduce susceptibility to the effects of air pollution. For example, consuming diets high in omega-3 fatty acids, even at levels as low as one fish meal per week, has been associated with lower mortality risk from CHD (Marchioli 2003). Although findings regarding supplementation are not entirely consistent, long-term supplementation with omega-3 fatty acids has also been shown to reduce the likelihood of nonfatal myocardial infarctions and stroke, as well as the risk of all-cause, cardiovascular, and sudden deaths (Marchioli 2003). As with omega-3 consumption, reducing salt intake can also contribute to cardiovascular health; studies suggest a 4–6 g/day reduction is associated with a reduction in systolic blood pressure of about 3.5 mmHg and 7 mmHg among normotensive and hypertensive (≥ 140/90 mmHg) individuals respectively, yielding a predicted avoidance of 240–362 cardiovascular events per 100,000 population over 10 years (He and MacGregor 2002; Qin et al. 2009). Consuming a diet high in plant sterols, combining four groups of cholesterol-lowering components of plant origin (viscous fibers, soy protein, plant sterols, and almonds), was also shown to significantly reduce blood pressure in a longitudinal study of 66 hyperlipidemic subjects (Jenkins et al. 2008).

Although literature regarding the effect of dietary modification on cardiovascular health is plentiful, there remains inconclusive evidence assessing how dietary modification or supplementation can modulate the effects of air pollution. Based on the currently prevalent notion that oxidative stress may play a role in the genesis of air pollution effects, it is logical to consider efforts to increase the body’s antioxidant defenses as potential interventions to ameliorate the negative health impacts of pollution exposure. Research investigating antioxidant supplementation suggests that intake of vitamins C and E can reduce the effects of ozone on lung function and nasal inflammatory cytokine production in both healthy and asthmatic populations (Chatham et al. 1987; Grievink et al. 1999; Samet et al. 2001; Sienra-Monge et al. 2004; Wiser et al. 2008). Omega-3 fatty acids, which can be found in oily fish such as mackerel and salmon and in flax seed oil, have also been studied because of their potential antioxidant benefit and ability to modulate the oxidative response to pollution. In a study of 52 older adults, daily supplementation with fish oil compared with soy oil reduced the effects of PM_2.5_ on superoxide dismutase activity, plasma glutathione, and heart rate variability (Romieu et al. 2005, 2008b). Similarly, analysis of supplementation with vitamins B_6_ or B_12_ or methionine in 549 elderly men as part of the Normative Aging Study suggests an attenuation of the effects of PM_2.5_ on heart rate variability (Baccarelli et al. 2008).

Taken together, these data suggest that supplementation with antioxidants such as vitamins C and E and omega-3 fatty acids, as well as vitamins B_6_ and B_12_ and methionine, can mitigate selected cardiovascular and respiratory impacts of ozone and PM. Furthermore, a limited body of research suggests that consuming omega-3 fatty acids, reducing salt intake, and having a predominantly vegetarian diet can reduce the baseline risk for the development of cardiovascular disease and could thereby reduce susceptibility to air pollution. Individuals who are considered at special risk of air pollution effects and those who wish to take positive action should be encouraged to follow more general dietary recommendations and increase consumption of fruits and vegetables. Specific recommendations regarding individual supplements and their dosing require substantial additional research.

### Medication

Although one study has indicated that drugs such as statins can abrogate an effect of PM exposure on heart rate variability in a subset of [glutathione *S*-transferase M1 (*GSTM1*) null] participants (Schwartz et al. 2005), the limited body of evidence is not sufficient at this point to justify specific recommendations for use of statins in relation to air pollution. Still, there are suggestions that optimal therapeutic management may protect individuals with inherent predisposition to altered heart rate variability from the effects of PM pollution: In a European study, evidence of altered heart rate variability in relation to PM exposure was strongest among study subjects not using beta-blocker medication, whereas effect modification was not evident for use of other medications (angiotensin-converting enzyme inhibitors, angiotensin receptor blockers, and statins) (de Hartog et al. 2009). Although the value of therapeutic management in mitigating the effects of pollutant exposure is worthy of further study, as are the potential benefits of dietary modification and supplementation, at present following evidence-based guidelines for primary and secondary prevention of cardiovascular disease is a reasonable approach to lowering the underlying baseline risk or air pollutant impacts.

### Modifying activity time, location, and level to reduce dose

Air quality advisories and ongoing air quality health information programs typically recommend changing the timing, location, duration, or intensity of outdoor activity to reduce short-term exposure and effective dose (Environment Canada 2010; U.S. Environmental Protection Agency 2010b), whereas optimal location of home, work, and school is the primary focus of advice related to long-term exposure (U.S. Environmental Protection Agency 2010a).

Air pollution dose is determined largely by the pollutant concentration and inhalation rate. Dose can be expressed over varying time windows (duration). Long-term exposures have been linked to increased risk for cardiovascular mortality (Dockery et al. 1993), whereas short-term exposures to peak pollutant levels have been associated with detrimental respiratory and cardiovascular effects. For example, a number of studies indicate that the incidence of ischemic heart disease events increases with recent exposure, perhaps even within 1–2 hr of exposure (Brook et al. 2010). Less evidence is available on the mechanistic effects of extremely short exposures in the range of minutes to hours. Extremely short-term exposures to high PM levels can occur in many situations, including in traffic jams, at bus stops, in indoor parking garages, and at fireworks displays (Dales et al. 2009; Glorennec et al. 2008; Singh et al. 2010). Short-term exposure to diesel exhaust (1–2 hr) significantly reduces brachial artery diameter in healthy subjects and exacerbates exercise-induced ST-segment depression in people with preexisting coronary artery disease (Mills et al. 2007a; Peretz et al. 2008). Exposure to PM may also alter pulmonary neural reflexes and lead to changes in heart rate variability through an increase in sympathetic stimulation, ultimately leading to arrhythmia (Brook et al. 2004). Although short-term exposure to PM has acute vascular and neural consequences, it is unclear how short-term exposures and specifically, repeated short-term exposures, affect health over the long term. At present there is insufficient data to assess the link between extremely short-term peak exposures and specific mechanisms of action leading to health outcomes. This important research gap makes it difficult to assess whether observed health effects are due to extremely short-term exposure to high concentrations of pollutants or to the average exposure over ≥ 1 days. An improved understanding of this relationship would inform better health recommendations, and messaging may need to be different depending on whether daily, 3-hr average or shorter peak exposures are the exposure duration of greatest consequence to health.

Pollutant levels may vary with time, depending on their sources. For example, fine PM levels may be higher during wintertime evenings in areas affected by residential wood burning, whereas ozone levels tend to be highest during summer afternoons, and traffic-related pollutants peak during rush hours. Human activity also has temporal patterns (Leech et al. 1996), and acute personal exposure to outdoor pollutants is greatest when peak times of outdoor activity correspond to peaks in ambient concentrations, as in the case of ozone in the afternoon (Liu et al. 1997). Personal exposures may be reduced severalfold by avoiding outdoor activity at peak times of day, although this may sometimes mean scheduling activity very early in the morning or deferring activity altogether on advisory days, depending on the pollutant (Campbell et al. 2005).

As described earlier, pollutant concentrations also vary in space. Individuals can reduce their exposure and the risk of adverse health effects by engaging in outdoor activity away from traffic (Kaur et al. 2005; McCreanor et al. 2007). Considering that some health effects, such as myocardial infarctions, may be associated with very short-term exposures, time spent in microenvironments where “high” exposures exist even for short durations, such as in a car during heavy traffic, can also be important (Mills et al. 2007b). Increased activity level increases inhalation rate up to severalfold, and those with higher activity levels in relation to work or recreation outdoors will receive a higher effective dose of pollution, particularly on days when outdoor air quality is poor. Reducing outdoor physical activity on days when air quality is particularly poor can decrease an individual’s air pollution dose, but it also reduces exercise levels. Although initial review of the literature suggests that beneficial aspects of active transportation outweigh any negative impacts related to increased air pollution exposure (de Hartog et al. 2010; de Nazelle and Nieuwenhuijsen 2010; Reynolds et al. 2010), further research is needed to better understand the health impacts of increased air pollution exposure during outdoor exercise; this will provide more balanced recommendations for individuals, which take into account the resulting benefits and risks. In addition, preliminary research suggests opportunities for planners that facilitate active transportation, without leading to increased air pollutant emissions or exposures (Su et al. 2010; Thai et al. 2008).

Given that most exposures, even to ambient pollution, occur indoors and that individuals may choose to remain or exercise indoors on days when outdoor air quality is poor, it is important that information on indoor air quality be included in health protection advice regarding air pollution exposure. Besides environmental tobacco smoke, which leads to well-documented exposures and effects, indoor sources such as cooking can generate high concentrations of PM indoors both in residences and commercial settings (Levy et al. 2002). The health impacts related to exposure to indoor-generated PM not related to smoking have not been thoroughly evaluated. Not surprisingly, following advice to stay indoors can reduce exposure to some pollutants while increasing exposure to others (Stieb et al. 2008). There is large variation in indoor:outdoor ratios for pollutant concentrations, both between and within homes. Indoor:outdoor pollutant ratios depend on numerous factors, including the type of pollutant, city, indoor and outdoor sources, building design, use of windows, age of the building, and season. Infiltration efficiency is the fraction of outdoor pollution that penetrates indoors and remains suspended and can be decreased by modifying penetration (the movement of outdoor pollutants to indoors), deposition (the depositing of pollutants on room surfaces), and exfiltration (the movement of pollutants to outdoors). Specifically, decreasing air exchange within a home or building can effectively reduce infiltration. Air conditioning and its coincidence with closing of windows, and the winter season (when windows are also generally closed) all function to reduce infiltration of ozone and PM by reducing air exchange (Allen et al. 2003; Liu et al. 1995; Wallace and Williams 2005).

Indoor pollutant exposure can also be lowered through the use of air cleaners. Several studies have shown that high-efficiency particulate air (HEPA) filter air cleaners can effectively reduce indoor PM concentrations resulting from both indoor (Batterman et al. 2005; Cheng et al. 1998; Green et al. 1999; Offermann et al. 1985) and outdoor (Barn et al. 2008; Brauner et al. 2008; Henderson et al. 2005) sources. However, these studies show that air cleaner effectiveness will differ within and between buildings depending on factors such as air exchange, the capacity of the air cleaner, and baseline pollutant levels. Clinical studies investigating the health benefits of air cleaner use have shown mixed results. A limited number of studies suggest that air cleaners can provide some health benefits by reducing exposure to PM that subsequently trigger biological responses associated with air pollutant exposure (Sublett et al. 2009). Some associations have been found between the use of HEPA filter devices and a reduction in asthma symptoms among adults and children (McDonald et al. 2002) and cat allergy–related symptoms among adults (van der Heide et al. 1997) associated with indoor-generated pollution and allergens. Researchers have also found associations between the use of portable air cleaners and decreased symptoms relating to exposure to outdoor generated pollution (Mott et al. 2002). In one study of elderly persons living in close proximity (< 350 m) to major roads, the use of two portable HEPA filter air cleaners over a 48-hr period was shown to decrease the impact of outdoor-generated PM on microvascular function (Brauner et al. 2008). In contrast, other studies have found no association between air cleaner use and air pollution–related health effects (Blackhall et al. 2003; Warburton et al. 1994; Wood et al. 1998). In a recent review, researchers suggested that investigating the health impacts of air cleaner use over a short-term period (days to weeks), as is the case for most studies, may not allow sufficient time to detect any resulting health benefits (Sublett et al. 2009). In addition to air cleaning, the use of air conditioning has been linked to some reduction in health impacts related to air pollution such as a decreased risk of cardiovascular hospitalizations in communities with a higher prevalence of air conditioning (Bell et al. 2009). The role of air conditioning is presumably related to reduced pollutant infiltration due to the decreased air exchange rates during the use of an air conditioner (because windows are closed), but the above ecologic association may also result from regional and/or socioeconomic factors and may not be specifically linked to air conditioner use (Vedal 2009).

## Research Gaps

### Mechanisms

To develop an optimal mix of community and individual actions, it is important to understand how long-term, short-term, and very short-term (subdaily, over the course of several hours) exposure to air pollution affects disease mechanisms and particularly disease progression and reversibility. A clearer understanding of this relationship could support the development of improved health messaging around exposure reduction, particularly among susceptible individuals.

Additionally, greater understanding of the underlying mechanisms related to factors such as diet and supplementation that may mitigate these health effects can provide a more substantial evidence base to inform decision making.

### Community interventions

Researching the health implications of the built environment and urban planning decisions should be a priority for public health. Specifically, further investigation of health outcomes associated with living, working, and exercising in locations in close proximity to major roads is needed. Within-city gradients of air pollution are particularly important to take into account; new modeling approaches are key for capturing the magnitude of within-city variations in air pollution.

To implement effective interventions that separate people from pollution, a greater understanding of exposure at the community and individual level is needed. Currently, many monitoring networks are sparsely located and do not provide information at the community or local scale, particularly for traffic-generated pollutants that are known to vary over small scales (Jerrett et al. 2005). Approaches that provide information at higher spatial resolution and account for factors such as topography could allow for better characterization of exposure to individuals as well as help to identify any potentially “high-exposure” situations that would benefit from targeted interventions; some of these approaches are reviewed by others (Hoek et al. 2008; Jerrett et al. 2005).

### Individual interventions

Further research is needed to document the effects of specific personal-level interventions on relevant clinical outcomes. For example, future research should consider long-term implications of exposure to air pollutants when determining the impact of diet and the impact of antioxidant supplementation, as well as the dose and duration of supplementation that would provide the greatest protective effect, in terms of clinically relevant outcomes.

Given the potential conflict between advice to reduce strenuous outdoor activity in order to avoid outdoor air pollution exposure, and the broader health benefits of physical activity, it is imperative to better understand the interaction of exercise and poor air quality on cardiorespiratory health and function in healthy, compromised, and athlete populations (Florida-James et al. 2004; Lippi et al. 2008). Although the recommendation of remaining indoors during high outdoor pollution events is supported by exposure data, no data on direct health benefits exist. Further evaluation needs to be conducted in order to better advise individuals on appropriate exposure reduction strategies. Additionally, a better understanding of the relative toxicity of indoor- and outdoor-generated pollutants, and the role of copollutants, both indoors and outdoors, in producing health effects, is needed. Finally, implementing and assessing strategies at indoor locations where individuals spend a lot of time would be beneficial. For example, providing and assessing the effects of air conditioning at schools located along major roadways would be useful.

## Summary

Regulatory interventions, which have been the primary focus of air quality management, are essential in reducing ambient pollutant levels and, consequently, health impacts among the public. To complement progress made through regulation, interventions implemented at the community and individual levels should also be given attention. Although there are some recent exceptions, such as California Senate Bill 352 (State of California 2003) regarding school locations in relation to roadways, land-use decisions typically do not consider air pollution–related health impacts.

Individual-level interventions that influence personal behaviors to modify pollutant exposure and/or dose are also potentially useful approaches to mitigate health effects of air pollution. Further, reducing individual baseline disease risk will mitigate air pollutant impacts on disease progression, whereas targeted interventions focused on diet, supplementation, and physical activity can reduce individual susceptibility to air pollution. Stressing individual-level interventions, however, raises the issues of burden of responsibility and environmental justice.

With this in mind, it is important to develop a new framework that approaches air quality and health from regulatory and community- and individual-level perspectives. Working within an evidence-based multidisciplinary public health framework and incorporating a stronger evidence base that addresses current knowledge gaps will allow us to move from good intentions to proven interventions.
